# Viabilidade do Implante de Eletrodo Ventricular Esquerdo na Terapia de Ressincronização Cardíaca Guiada por Gated SPECT e Remodelamento Ventricular

**DOI:** 10.36660/abc.20220077

**Published:** 2023-03-20

**Authors:** Erivelton Alessandro do Nascimento, Fernando de Amorim Fernandes, Pedro Augusto Carvalho Mira, Zhuo He, Weihua Zhou, Claudio Tinoco Mesquita

**Affiliations:** 1 Universidade Federal Fluminense Hospital Universitário Antônio Pedro Pós-Graduação em Ciências Cardiovasculares Niterói RJ Brasil Universidade Federal Fluminense – Serviço de Cardiologia, Hospital Universitário Antônio Pedro e Pós-Graduação em Ciências Cardiovasculares, Niterói, RJ – Brasil; 2 Instituto Estadual de Cardiologia Aloysio de Castro Departamento de Arritmias Cardíacas Rio de Janeiro RJ Brasil Instituto Estadual de Cardiologia Aloysio de Castro – Departamento de Arritmias Cardíacas, Rio de Janeiro, RJ – Brasil; 3 Universidade Federal Fluminense Departamento de Radiologia Pós-Graduação em Ciências Cardiovasculares Niterói RJ Brasil Universidade Federal Fluminense – Departamento de Radiologia – Universidade Federal Fluminense e Pós-Graduação em Ciências Cardiovasculares, Niterói, RJ – Brasil; 4 Universidade Federal Fluminense Laboratório de Ciências do Exercício Niterói RJ Brasil Universidade Federal Fluminense – Laboratório de Ciências do Exercício, Niterói, RJ – Brasil; 5 Department of Applied Computing Michigan Technological University Houghton MI EUA Department of Applied Computing, Michigan Technological University, Houghton – MI – EUA

**Keywords:** Insuficiência Cardíaca, Terapia de Ressincronização Cardíaca, SPECT, Dissincronia Ventricular Esquerda

## Abstract

**Fundamento:**

A terapia de ressincronização cardíaca (TRC) pode beneficiar pacientes com insuficiência cardíaca (IC) avançada. O índice de excentricidade anormal por gated SPECT está relacionado a alterações estruturais e funcionais do ventrículo esquerdo (VE).

**Objetivo:**

O objetivo do presente estudo foi avaliar a viabilidade do implante de eletrodos do VE guiado por análise de fase e sua relação com o remodelamento ventricular.

**Métodos:**

Dezoito pacientes com indicação de TRC foram submetidos à cintilografia miocárdica para orientar o implante, avaliando-se os parâmetros de excentricidade e forma ventricular. P < 0,05 foi adotado como significância estatística.

**Resultados:**

Na linha de base do estudo, a maioria dos pacientes foi classificada como NYHA 3 (n = 12). Após a TRC, 11 dos 18 pacientes foram reclassificados para um menor grau de limitação funcional. Além disso, a qualidade de vida dos pacientes melhorou após a TRC. Foram observadas reduções significativas na duração do QRS, intervalo PR, índice de forma diastólica final, índice de forma sistólica final, volume sistólico e massa miocárdica pós-TRC. O eletrodo do VE da TRC foi posicionado concordante, adjacente e discordante em 11 (61,1%), 5 (27,8%) e 2 (11,1%) pacientes, respectivamente. A excentricidade sistólica e diastólica final demonstrou remodelamento reverso após a TRC.

**Conclusões:**

O implante de eletrodo do VE em TRC guiado por cintilografia gated SPECT é viável. A colocação do eletrodo concordante ou adjacente ao último segmento a se contrair foi um determinante do remodelamento reverso.

## Introdução

A insuficiência cardíaca (IC) afeta mais de 5 milhões de pessoas nos Estados Unidos. Cerca de 550.000 novos casos são diagnosticados anualmente, e a IC descompensada é responsável por mais de 1 milhão de internações por ano.^1^ As projeções mostram que a prevalência de IC aumentará em 46% de 2012 a 2030, resultando em mais de 8 milhões de indivíduos acima de 18 anos de idade com IC.^2^ Como resultado dessa transição epidemiológica, dos avanços nos cuidados de saúde e do envelhecimento da população, a prevalência de doença arterial coronariana, hipertensão arterial sistêmica, obesidade e diabetes mellitus está aumentando e terá um impacto significativo na incidência de IC em países em desenvolvimento.^3^ A terapia de ressincronização cardíaca (TRC) tornou-se uma opção de tratamento para IC sintomática em que a disfunção ventricular esquerda (VE), dissincronismo elétrico e terapia clínica otimizada estão presentes. Essa técnica mostrou uma melhora significativa na classe funcional da New York Heart Association (NYHA) e na fração de ejeção em pacientes com disfunção ventricular grave e bloqueio de ramo esquerdo.^4^ No entanto, um grupo significativo de pacientes não responde favoravelmente à TRC.^5-7^ Os pacientes com doença arterial coronariana e histórico de infarto do miocárdio são menos propensos a responder, devido à presença de fibrose. Portanto, os critérios de seleção atualmente usados não parecem ser os ideais, considerando que, em estudos anteriores de TRC usando esses critérios, uma porcentagem significativa de pacientes (20% a 40%) não se beneficiou da terapia.^5-7^ O eletrocardiograma tem sido usado como método para detectar pacientes com dissincronismo devido à correlação entre o alargamento do complexo QRS (dissincronismo elétrico) e a presença de dissincronismo mecânico. Portanto, é de grande valia estudar o sincronismo ventricular previamente à TRC para estimar a sua resposta, por ser este último um procedimento de alto custo. A análise de fase para avaliação da dissincronia do VE foi incorporada pela cintilografia de perfusão miocárdica com gated SPECT (tomografia computadorizada por emissão de fóton único).^8^ A análise de fases permite avaliar, além dos parâmetros de sincronismo, o último segmento ventricular a se contrair, de forma altamente reprodutível.^9-13^ Os pacientes com bloqueio de ramo esquerdo tendem a ter o início da contração mecânica do VE mais cedo no ciclo cardíaco na parede septal e mais tarde em outras regiões do miocárdio por causa da desaceleração da propagação do impulso nervoso pelo sistema de condução, causando uma ativação tardia, sendo o último local de contração mais comumente localizado na parede póstero-lateral. Um estudo em modelo experimental na década de 1990 demonstrou que o início da regurgitação mitral funcional é acompanhado por alterações geométricas do VE, manifestadas por aumento da esfericidade.^14^ Um índice de excentricidade anormal é um marcador de remodelamento adverso na IC. As anormalidades do índice de excentricidade pelo gated SPECT estão relacionadas a alterações estruturais e funcionais do VE.^15^ No presente estudo, avaliamos a viabilidade do implante de eletrodos no VE guiado pela cintilografia miocárdica com gated SPECT e suas implicações para o remodelamento do VE.

## Métodos

### Desenho do estudo

No presente estudo, realizamos amostragem consecutiva, composta por 20 pacientes com IC que foram incluídos prospectivamente para implante de um dispositivo de TRC. Após preencher o termo de consentimento, todos os pacientes foram submetidos ao eletrocardiograma de 12 derivações e responderam ao Minnesota Living with Heart Failure Questionnaire (MLHFQ) imediatamente antes do estudo de cintilografia gated SPECT e subsequente implante do dispositivo, e repetiram todas essas etapas 6 ± 1 meses após implante de ressincronizador.

### Critérios de inclusão

O presente estudo contém dados nacionais que fazem parte do estudo multicêntrico internacional VISION CRT,^16^ cujos dados foram previamente publicados. O estudo incluiu pacientes estáveis com mais de 18 anos de idade, com classe funcional NYHA II a IV por pelo menos 3 meses antes da inclusão no estudo, apesar de receber tratamento médico otimamente tolerado de acordo com as diretrizes atuais (incluindo doses estáveis de um inibidor da enzima conversora de angiotensina ou antagonista dos receptores de angiotensina, antagonistas dos receptores mineralocorticoides e um betabloqueador por pelo menos 3 meses). Os critérios de inclusão foram os seguintes: fração de ejeção do ventrículo esquerdo (FEVE) ≤ 35% por causas isquêmicas ou não isquêmicas, medida conforme procedimento usual no centro participante; duração intrínseca do QRS ≥ 120 ms, com morfologia de bloqueio de ramo esquerdo; ritmo sinusal; consentimento informado por escrito; pacientes com implante de cardiodesfibrilador implantável para prevenção primária ou secundária de morte súbita cardíaca.

### Critérios de exclusão

Foram excluídos deste estudo pacientes com fibrilação atrial ou flutter atrial, doença grave, sobrevida inferior a um ano, bloqueio de ramo direito, gravidez ou amamentação ou síndromes coronarianas agudas.

### Técnicas do procedimento

#### Cintilografia de perfusão miocárdica com gated SPECT

A aquisição e a reconstrução das imagens e o controle de qualidade do equipamento foram realizados conforme indicado a seguir: análise e processamento de imagens usando o software Emory Cardiac Toolbox, versão 4 (ECTb4). Atividade: ~ 10 a 20 mCi (ajustado pelo peso 0,2 mCi/kg [somente estudo em repouso]), não ultrapassando 20 mCi, em decúbito dorsal. O radiotraçador utilizado foi o Tc-99-sestamibi.

#### Protocolo de aquisição

Atraso da imagem da injeção: 45 a 60 min; janela de energia: 15% a 20% simétrica; colimador: baixa energia, alta resolução; órbita: 180° (45° oblíquo anterior direito a 45° oblíquo posterior esquerdo); tipo de órbita: circular; tamanho de pixel: 6,4 ± 0,4 mm; tipo de aquisição: *step-and-shoot*; número de projeções: ≥ 60; matriz: 64 × 64 e 128 × 128 (opcional); tempo/projeção (gama de 2 cabeças c): 20 segundos; tempo/projeção (gama de 1 cabeça c): 30 segundos (com 20 mCi); ECG gated, quadros/ciclo: 8 padrão e 16 (opcional); janela R-para-R: 100%.

#### Processamento de imagem

Foram realizadas reconstruções FBP e OSEM com um filtro equivalente a um Butterworth (ordem 10 e frequência de corte 0,4 ciclo/pixel). Foram analisadas as imagens de gated SPECT em repouso usando a análise de fase 1-harmônica para medir a dissincronia sistólica do VE, incluindo o desvio padrão (DP) da fase sistólica. O laboratório central avaliou as regiões de fibrose cicatricial e indicou a última região viável do VE a se contrair para análise da colocação concordante do eletrodo do VE.

#### Processamento do laboratório nuclear central

O controle de qualidade foi realizado em relação à densidade de contagem de aquisição e gating, bem como à adequação da reconstrução. O ECTb4 foi usado para medir automaticamente o volume sistólico final do ventrículo esquerdo (VSFVE), o volume diastólico final do ventrículo esquerdo (VDFVE), FEVE, DP de fase e local da última ativação mecânica tanto para o estudo de linha de base quanto para o estudo de acompanhamento de 6 meses. Todo o processamento passou por controle de qualidade após o processamento automático para confirmar a determinação correta do ângulo oblíquo do VE, base, ápice e posição do cursor do centro do VE. O local da última ativação mecânica foi determinado usando o modelo de 6 segmentos (septal, ântero-septal, anterior, lateral, posterior e inferior). As regiões de interesse correspondentes ao modelo de 6 segmentos foram colocadas automaticamente na distribuição de fase tridimensional (mapa polar). Cada região de interesse abrange 45° e 6 fatias de eixo curto começando pela fatia do meio em direção à base. Como há uma amostra miocárdica a cada 9°, cada região de interesse conterá 30 (5 × 6) amostras. As fases médias dos 6 segmentos foram calculadas pela média das fases de suas 30 amostras e então comparadas. O último segmento ativado mecanicamente foi aquele com os maiores ângulos de fase médios. O VSFVE, VDFVE, FEVE, DP de fase e local da última ativação mecânica para o estudo de linha de base e o estudo de acompanhamento de 6 meses foram relatados por meio de um site SharePoint usando o formulário de análise de laboratório nuclear central.

#### Protocolo de TRC

Os pacientes foram selecionados para implante de TRC. Para avaliar a posição do eletrodo VE, as imagens foram registradas em fluoroscopia usando orientação oblíqua anterior esquerda de 40° com inclinação caudal e posição oblíqua anterior direita de 30° para obter a melhor separação das veias do seio coronário.

#### Determinação da colocação do eletrodo

Em todos os pacientes, a posição final do eletrodo do VE foi determinada e categorizada como basal-ou-média ou apical no segmento anterior, lateral, posterior, inferior e septal ou ântero-septal (improvável). A colocação do eletrodo foi classificada como: concordante (no último segmento), adjacente (até um segmento do último) e discordante (mais de um segmento do último).^17^

## Análise estatística

As variáveis categóricas foram apresentadas como números absolutos (porcentagem) e as variáveis contínuas foram apresentadas como média e DP ou mediana e intervalo interquartílico, de acordo com a normalidade dos dados. Foi utilizado o teste de Shapiro-Wilk para analisar as distribuições dos dados. As comparações entre pré e pós-TRC foram realizadas usando o teste t de Student para amostras pareadas ou o teste de Wilcoxon. P < 0,05 foi adotado como significância estatística. Todas as análises foram realizadas no programa SPSS, versão 20.0 (IBM Corp., NY, EUA).

## Resultados

Após a inclusão, 2 pacientes faleceram por causas não cardiológicas, antes de completarem as etapas de avaliação, e 18 participantes completaram o protocolo. As características dos participantes são apresentadas na Tabela 1.

**Tabela 1:** – Características dos participantes

Variáveis	Pacientes (n = 18)
Idade (anos)	65 ± 7
Altura (m)	1,36 ± 0,09
Peso (kg)	70,65 ± 16,45
Doença arterial coronariana, n (%)	8 (44,4)
Diabetes, n (%)	7 (38,9)
**Raça, n (%)**	
Branca	5 (27,8)
Negra	9 (50,0)
Outras	4 (22,2)

*Os dados são apresentados como média ± desvio padrão ou número de pacientes (porcentagem).*

**Figura 1:**
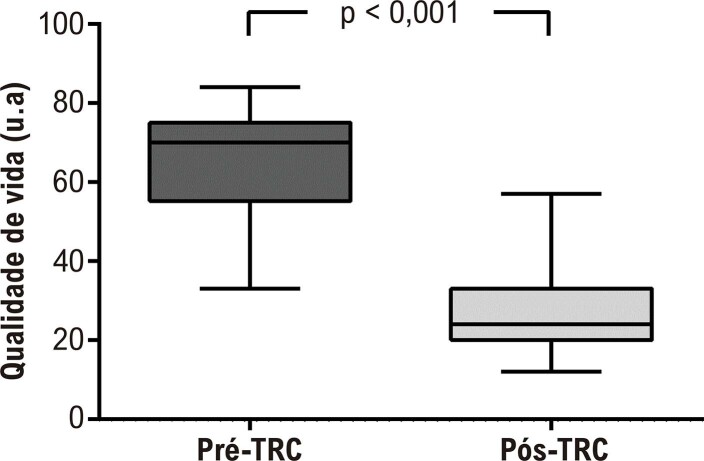
– Qualidade de vida pré e pós-terapia de ressincronização cardíaca (TRC). u.a: unidade absoluta.

Na linha de base do estudo, a maioria dos pacientes foi classificada como NYHA III (n = 12), seguida de NYHA IV (n = 5) e NYHA II (n = 1). Após a TRC, 11 dos 18 pacientes foram reclassificados para um grau menor de limitação funcional, e nenhum dos pacientes foi classificado como NYHA IV. Todos os 5 pacientes classificados como NYHA IV e 7 dos pacientes classificados como NYHA III pré-CRT foram reclassificados como NYHA II pós-CRT. Além disso, a qualidade de vida dos pacientes melhorou após a TRC (Figura 1). Em nossa amostra, 44,4% dos pacientes apresentavam etiologia isquêmica; no entanto, a carga de fibrose estava abaixo de 40%.

As variáveis do eletrocardiograma e SPECT são apresentadas na Tabela 2. A TRC aumentou a FEVE, a excentricidade diastólica final (EDF) e a excentricidade sistólica final (ESF). Em contraste, foram observadas reduções significativas na duração do QRS, intervalo PR, índice de forma diastólica final, índice de forma sistólica final, volume sistólico e massa miocárdica pós-TRC. Além disso, após a TRC, também foi reduzido o índice de volume diastólico final.

**Tabela 2:** – Variáveis do eletrocardiograma e SPECT

Variáveis	Pré-CRT	Pós-CRT	p
Duração do QRS (ms)	194,83 ± 23,68	119,78 ± 11,65	< 0,001
Intervalo PR (ms)	190,0 (167,5 – 210,0)	90,0 (90,0 – 100,0)	< 0,001
IVDF (ml/m^2^)	202,5 (175,0 – 281,75)	164,0 (110,0 – 277,0)	0,058
IVSF (ml/m^2^)	165,5 (124,0 – 209,25)	108,0 (61,5 – 242,75)	0,157
IFDF	0,87 ± 0,11	0,76 ± 0,14	0,001
IFSF	0,80 ± 0,08	0,71 ± 0,12	0,004
FEVE (%)	28,11 ± 5,93	40,94 ± 11,09	0,001
EDF	0,5 ± 0,1	0,6 ± 0,2	0,040
ESF	0,6 ± 0,1	0,7 ± 0,1	0,041
Volume sistólico (ml)	66,50 (54,75 – 83,75)	51,00 (48,75 – 61,00)	0,002
LBF (grau)	58,5 (39,5 – 108,3)	68 (65 – 72,25)	0,777
DPF (grau)	23,5 (13,4 – 43,1)	21,1 (20,0 – 26,6)	0,372
SPECT, pico de fase	120,33 ± 34,25	118,22 ± 28,19	0,836
SPECT, inclinação de fase	3,00 (2,86 – 3,37)	2,95 (2,33 – 3,45)	0,231
SPECT, curtose de fase	10,61 (8,05 – 14,42)	8,76 (5,83 – 17,50)	0,586
Massa miocárdica (g)	207,5 (185,0 – 262,5)	143,5 (137,25 – 208,25)	0,004

*Os dados são apresentados como média ± desvio padrão ou mediana (percentis 25 a 75). DPF: desvio padrão de fase, EDF: excentricidade diastólica final, ESF: excentricidade sistólica final, FEVE: ventrículo esquerdo fração de ejeção, IFDF: índice de forma diastólica final, IFSF: índice de forma sistólica final, IVDF: índice de volume diastólico final, IVSF: índice de volume sistólico final, LBF: largura de banda de fase.*

Analisando as variáveis cintilográficas após a TRC, observou-se um aumento da curtose da fase diastólica por SPECT. As demais variáveis permaneceram inalteradas após a TRC.

**Figura 2:**
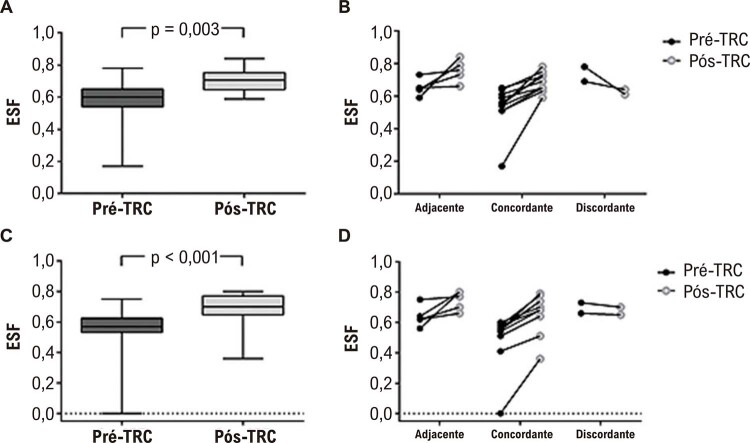
– Box plot e dados individuais comparando-se pré e pós-TRC (painéis A e C) e considerando se a posição do eletrodo do ventrículo esquerdo da TRC era adjacente, concordante ou discordante (painéis B e D). O box plot mostra a mediana (linha horizontal dentro da caixa), mínimo, percentis 25 a 75 e máximo. EDF: excentricidade diastólica final; ESF: excentricidade sistólica final; TRC: terapia de ressincronização cardíaca.

ESF e EDF são representados na Figura 2. O eletrodo do VE da TRC foi posicionado concordante, adjacente e discordante em 11 (61,1%), 5 (27,8%) e 2 (11,1%) pacientes, respectivamente. ESF e EDF aumentaram pós-CRT (Figura 2A e 2C, respectivamente). Dados individuais mostraram que ESF (Figura 2B) e EDF (Figura 2D) aumentaram tanto no posicionamento adjacente quanto no concordante. Em contraste, essas variáveis diminuíram apenas nos 2 pacientes em que a posição do eletrodo do VE da TRC foi discordante em relação ao último segmento a se contrair.

**Figura 3:**
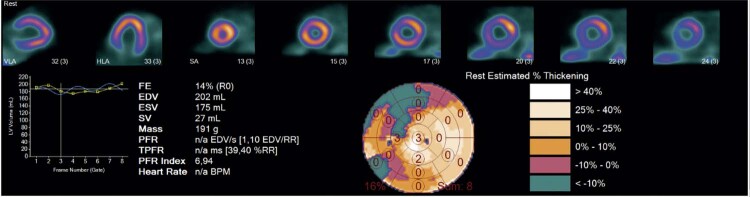
– Paciente de 64 anos antes da TRC, com insuficiência cardíaca de origem hipertensiva e disfunção grave do VE. FE: 14%, EDF: 0,56, ESF: 0,59, IFSF: 0,81, IFDF: 0,83, VSFVE: 175 ml, massa do VE: 191 g. EDF: excentricidade diastólica final, ESF: excentricidade sistólica final, FE: fração de ejeção, IFDF: índice de forma diastólico final, IFSF: índice de forma sistólico final, VSFVE: volume sistólico final do ventrículo esquerdo.

A Figura 3 mostra a largura de banda do histograma (HBW, do inglês *histogram bandwidth*) na análise de fase antes da TRC, demonstrando importante dissincronia.

**Figura 4:**
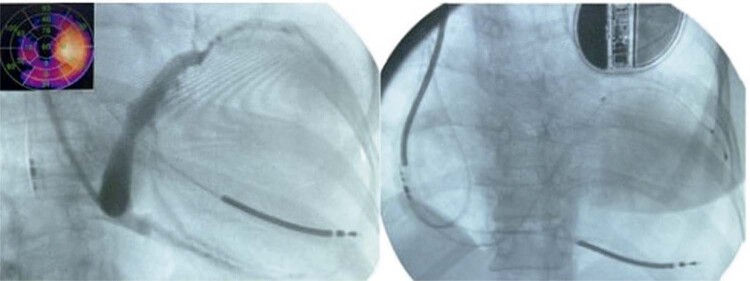
– Fluoroscopia durante a implantação do dispositivo (TRC-CDI). A imagem da esquerda mostra a venografia do seio coronário e suas tributárias e mapa polar. A imagem da direita mostra o posicionamento final dos eletrodos de choque (ventrículo direito) e dos eletrodos do átrio direito e do ventrículo esquerdo em segmento concordante, posicionados no seio coronário.

A Figura 4 mostra as imagens do procedimento de implantação do dispositivo no local de maior atraso.

**Figura 5:**
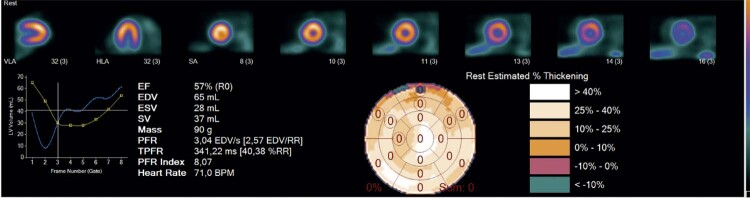
– O paciente da Figura 3 demonstrando super-resposta após a TRC. FE: 57%, ESF: 0,84, EDF: 0,80, IFSF: 0,54 IFDF: 0,6, VSFVE: 28 ml e massa do VE: 90 g. EDF: excentricidade diastólica final, ESF: excentricidade sistólica final, FE: fração de ejeção, IFDF: índice de forma diastólico final, IFSF: índice de forma sistólico final, VSFVE: volume sistólico final do ventrículo esquerdo.

Na Figura 5, um paciente demonstra super-resposta à TRC 6 meses após o procedimento.

## Discussão

A dissincronia do VE tem sido avaliada por várias modalidades de imagem cardiovascular, incluindo ecocardiograma com Doppler tecidual ou mesmo strain^18,19^ ou ressonância magnética;^20^ e imagem nuclear por meio de ventriculografia radioisotópica ou SPECT.^21^ A contração do VE foi inicialmente analisada com sucesso por ventriculografia radionuclídica, mas, com a adição de análise de fase para gated SPECT e sua subsequente validação, esta técnica tem demonstrado excelente potencial na determinação da dissincronia mecânica do VE. A gated SPECT permite a avaliação da dissincronia do VE usando as funções harmônicas de Fourier para estimar o espessamento da parede ao longo do ciclo cardíaco e determinar o momento do início regional da contração mecânica do ventrículo, obtendo uma análise quantitativa tridimensional de todo o VE.

A análise de fase da cintilografia de perfusão miocárdica usa 2 variáveis principais para prever a resposta à TRC. Os valores de corte de 135º para alargamento da banda (HBW) e 43º para o DP podem predizer a resposta clínica.^8,21,22^ No presente estudo, encontramos uma média de 92,5º e 31º antes da ressincronização para HBW e DP de fase, respectivamente.

Além dos parâmetros de dissincronia, a análise de fase permite avaliar o último segmento do VE a se contrair de forma altamente reprodutível. Os pacientes com bloqueio de ramo esquerdo tendem a ter o início da contração mecânica do VE mais cedo no ciclo cardíaco na parede septal e mais tarde em outras regiões do miocárdio por causa da desaceleração da propagação do impulso nervoso pelo sistema de condução, causando uma ativação tardia, sendo o último local de contração mais comumente localizado na parede inferior ou lateral.^9^ Estudos que realizaram implante de eletrodos do VE de acordo com os achados do último segmento a se contrair pela análise de fase por gated SPECT demonstraram melhora clínica significativa.^9^ Os parâmetros que indicam alteração aguda do sincronismo após a TRC são: (a) presença de dissincronia basal definida por DP e HBW > 2 DP acima dos limites normais, (b) presença de fibrose < 40% do VE e (c) concordância da posição do eletrodo, definida como a colocação do eletrodo do VE no último segmento a se contrair, com base no mapa polar.^10,23^ Essa variável foi viável no presente estudo, onde o implante do eletrodo colocado de acordo com o último segmento a se contrair foi alcançado em aproximadamente 60% dos pacientes.

Segundo achados anteriores, a hipertrofia excêntrica do VE é variável independente para morte súbita cardíaca arritmogênica,^24^ variável presente em todos os pacientes do estudo, somada à FEVE abaixo de 35% e à doença arterial coronariana em 44% dos participantes.

Do mesmo modo observado em um estudo anterior, a maioria dos pacientes do presente estudo (n = 17; 94,4%) apresentaram para terapia de ressincronização em classe funcional da NYHA III ou IV.^25^ Os achados de melhora da qualidade de vida, avaliados pelo MLHFQ, foram significativos. As avaliações clínico-funcionais do presente estudo corroboram o benefício da TRC já observado em múltiplos estudos.^25-27^ Apesar de avaliar dados subjetivos, o MLHFQ refere-se à percepção do paciente sobre seus sintomas, e o estudo InSync descreveu essa percepção de melhora avaliada pelo MLHFQ.^28^ Nascimento et al. demonstraram melhora da qualidade de vida e relação entre sincronismo eletromecânico e resposta à TRC na posição do eletrodo do VE guiado por gated SPECT.^29^

Os nossos achados estão de acordo com os dados de He et al.,^30^ onde os parâmetros da geometria do VE obtidos pelo gated SPECT foram capazes de predizer a super-resposta à TRC associada à orientação da colocação do eletrodo do VE pelo gated SPECT, no qual observamos uma alteração significativa da variável de excentricidade do VE tanto na sístole quanto na diástole do VE, denotando o remodelamento reverso após a TRC guiada por gated SPECT. Um achado significativo em nossos dados é que, na posição discordante do eletrodo do VE em 2 pacientes, a variável de excentricidade comportou-se de forma diferente das demais posições, não levando ao remodelamento reverso, com piora da geometria do VE.

Estudos recentes relataram a presença de defeitos de perfusão ou de tecido cicatricial e a influência na resposta à TRC,^31,32^ e o gated SPECT tem uma vantagem nessa abordagem, pois permite integrar avaliação da função do VE, da perfusão (para identificar isquemia e tecido cicatricial) e da dissincronia.

O acompanhamento do estudo Imaging CRT que usou multimodalidade para guiar o implante de eletrodos do VE na TRC não mostrou redução no resultado composto de hospitalizações por IC e mortalidade por todas as causas.^33^ A classificação do remodelamento do VE baseada na espessura relativa da parede e na massa ventricular nos leva à ideia de que, na remodelação excêntrica, existe dilatação da câmara do VE sem aumento da massa do VE.^34^

Os dados do estudo MIRACLE de remodelação reversa sustentada demonstraram uma redução sustentada na massa ventricular aos 6 e aos 12 meses após a TRC. Conforme observado em nossos dados, a massa miocárdica teve redução significativa na análise pré-TRC e 6 meses após a TRC, de 207,5 g e 143,5 g, respectivamente.^35^

O presente estudo apresenta algumas limitações, dentre elas: a inclusão de um número relativamente pequeno de pacientes; a não randomização dos pacientes para um grupo controle; o curto período de seguimento, que pode influenciara avaliação do comportamento das variáveis do remodelamento ventricular; e a não utilização de eletrodos quadripolares, que poderia ampliar a avaliação da colocação dos eletrodos do VE.

Futuros estudos randomizados com maior número de pacientes são necessários para melhor avaliar a correlação entre o implante do eletrodo do VE e o remodelamento ventricular na TRC.

## Conclusão

O implante de eletrodos do VE em TRC guiado por cintilografia gated SPECT é viável. A colocação do eletrodo concordante ou adjacente ao último segmento a se contrair foi um determinante da resposta à TRC, levando ao remodelamento reverso avaliado pelo índice de excentricidade do VE, e essa colocação foi alcançada em 88,9% dos pacientes no presente estudo. Futuros estudos prospectivos são necessários em populações maiores.
