# Supplementary material for: Viabilidade do Implante de Eletrodo Ventricular Esquerdo na Terapia de Ressincronização Cardíaca Guiada por Gated SPECT e Remodelamento Ventricular
Source: Arq Bras Cardiol. 2023 Mar 20;120(3):e20220077. [Article in Portuguese] doi: 10.36660/abc.20220077 (PMC10392842; doi:10.36660/abc.20220077)
Supplement: Translation

# Translation: Feasibility of Left Ventricle Lead Implantation in Cardiac Resynchronization Therapy Guided by Gated SPECT and Ventricular Remodeling

**Authors:** Erivelton Alessandro do Nascimento, Fernando de Amorim Fernandes, Pedro Augusto Carvalho Mira, Zhuo He, Weihua Zhou, Claudio Tinoco Mesquita

**Affiliations:** 1 Brasil Universidade Federal Fluminense – Serviço de Cardiologia, Hospital Universitário Antônio Pedro e Pós-Graduação em Ciências Cardiovasculares, Niterói, RJ – Brasil; 2 Brazil Instituto Estadual de Cardiologia Aloysio de Castro – Departamento de Arritmias Cardíacas, Rio de Janeiro, RJ – Brazil; 3 Brazil Universidade Federal Fluminense – Departamento de Radiologia – Universidade Federal Fluminense e Pós-Graduação em Ciências Cardiovasculares, Niterói, RJ – Brazil; 4 Brazil Universidade Federal Fluminense – Laboratório de Ciências do Exercício, Niterói, RJ – Brazil; 5 USA Department of Applied Computing, Michigan Technological University, Houghton – MI – USA

**Keywords:** Heart Failure, Cardiac Resynchronization Therapy, SPECT, Left Ventricular Dyssynchrony

## Abstract

**Background:**

Cardiac resynchronization therapy (CRT) may benefit patients with advanced heart failure (HF). Abnormal eccentricity index by gated SPECT is related to structural and functional alterations of the left ventricle (LV).

**Objective:**

The aim of this study is to evaluate the feasibility of LV lead implantation guided by phase analysis and its relationship to ventricular remodeling.

**Methods:**

Eighteen patients with indication for CRT underwent myocardial scintigraphy for implant orientation, and eccentricity and ventricular shape parameters were evaluated. P < 0.05 was adopted as statistical significance.

**Results:**

At baseline, most patients were classified as NYHA 3 (n = 12). After CRT, 11 out of 18 patients were reclassified to a lower degree of functional limitation. In addition, patients’ quality of life was improved post-CRT. Significant reductions were observed in QRS duration, PR interval, end-diastolic shape index, end-systolic shape index, stroke volume, and myocardial mass post-CRT. The CRT LV lead was positioned concordant, adjacent, and discordant in 11 (61.1%), 5 (27.8%), and 2 (11.1%) patients, respectively. End-systolic and end-diastolic eccentricity demonstrated reverse remodeling post-CRT.

**Conclusions:**

LV lead implantation in CRT guided by gated SPECT scintigraphy is feasible. The placement of the electrode concordant or adjacent to the last segment to contract was a determinant of reverse remodeling.

## Introduction

Heart failure (HF) affects more than 5 million people in the United States. About 550,000 new cases are diagnosed annually, and decompensated HF is responsible for more than 1 million admissions per year.^1^ Projections show that the prevalence of HF will increase by 46% from 2012 to 2030, resulting in more than 8 million individuals over 18 years of age with HF.^2^ As a result of this epidemiological transition, advances in health care and the aging of the population, the prevalence of coronary artery disease, systemic arterial hypertension, obesity, and diabetes mellitus is increasing and will have a significant impact on the incidence of HF in developing countries.^3^ Cardiac resynchronization therapy (CRT) has become a treatment option for symptomatic HF in which left ventricular (LV) dysfunction, electrical dyssynchrony, and optimized clinical therapy are present. This technique showed a significant improvement in the New York Heart Association (NYHA) functional class and ejection fraction in individuals with severe ventricular dysfunction and left bundle branch block^4^ However, a significant group of patients does not respond favorably to CRT.^5-7^ Patients with coronary artery disease and a history of myocardial infarction are less likely to respond, due to the presence of fibrosis. Therefore, the selection criteria currently used do not seem to be ideal, as in previous studies of CRT using these criteria, a significant percentage of patients (20% to 40%) did not benefit from therapy.^5-7^ The electrocardiogram has been used as a method for detecting patients with dyssynchronism due to the correlation between the widening of the QRS complex (electrical dyssynchronism) and the presence of mechanical dyssynchronism. Therefore, it is of great value to study ventricular synchronism prior to CRT in order to estimate its response, as this is a procedure that involves high costs. The phase analysis for the assessment of LV dyssynchrony was incorporated by myocardial perfusion scintigraphy with gated single-photon emission computed tomography (SPECT).^8^ Phase analysis makes it possible to assess, in addition to synchronism parameters, the last ventricular segment to contract, in a highly reproducible way.^9-13^ Patients with left bundle branch block tend to have the onset of mechanical contraction of the LV earlier in the cardiac cycle in the septal wall, and later in other regions of the myocardium because of the deceleration of the propagation of the nerve impulse by the conduction system, causing a late activation, with the last site of contraction most commonly located on the posterolateral wall. A study in an experimental model in the 1990s demonstrated that the onset of functional mitral regurgitation is accompanied by geometric changes in the LV, manifested by an increase in sphericity.^14^ Abnormal eccentricity index is a marker of adverse remodeling in HF. Abnormalities of the eccentricity index by gated SPECT are related to structural and functional alterations of the LV.^15^ In the present study, we evaluated the feasibility of LV lead implantation guided by gated SPECT myocardial scintigraphy and its implications for LV remodeling.

## Methods

### Study design

In this study we performed consecutive sampling, consisting of 20 patients with HF who were prospectively included for implantation of a CRT device. After completing the consent form, all patients underwent a 12-lead electrocardiogram and responded to the MLHFQ Minnesota Living with Heart Failure Questionnaire (MLHFQ) immediately before the gated SPECT scintigraphy study and subsequent device implantation, and repeated all these steps 6 ± 1 months after resynchronizer implantation.

### Inclusion criteria

The present study contains national data that are part of the international multicenter study VISION CRT,^16^ whose data were previously published. The study included stable patients over 18 years of age, with NYHA functional classes II to IV for at least 3 months before admission to the study, despite receiving optimally tolerated medical treatment according to current guidelines (including stable doses of an angiotensin converting enzyme inhibitor or angiotensin receptor antagonist, mineralocorticoid receptor blockers, and a beta-blocker for at least 3 months). The inclusion criteria were as follows: left ventricular ejection fraction (LVEF) ≤ 35% due to ischemic or non-ischemic causes, measured according to the usual procedure at the participating center; intrinsic QRS duration of ≥ 120 ms, with left bundle branch block morphology; sinus rhythm; consent informed in writing; patients with ICD implantation for primary or secondary prevention of sudden cardiac death.

### Exclusion criteria

Patients with atrial fibrillation or atrial flutter, serious illness, survival of less than one year, right branch block, pregnancy or breastfeeding, or acute coronary syndromes were excluded from this study.

### Procedure techniques

#### Gated SPECT myocardial perfusion scintigraphy

The acquisition and reconstruction of images and the quality control of the equipment were carried out as follows: image analysis and processing using Emory Cardiac Toolbox software version 4 (ECTb4). Activity: ~ 10 to 20 mCi (adjusted by weight 0.2 mCi/kg [rest study only]), not exceeding 20 mCi, in supine position. The radiotracer used was Tc-99-sestamibi.

#### Acquisition protocol

Imaging delay from injection: 45 to 60 min; energy window: 15% to 20% symmetric; collimator: low-energy, high-resolution; orbit: 180° (45° right anterior oblique to 45° left posterior oblique); orbit type: circular; pixel size: 6.4 ± 0.4 mm; acquisition type: step-and-shoot; number of projections: ≥ 60; matrix: 64 × 64 and 128 × 128 (optional); time/projection (2 head-gamma c): 20 seconds; time/projection (1 head-gamma c): 30 seconds (with 20 mCi); ECG gated, frames/cycle: 8 standard and 16 (optional); R-to-R window: 100%.

#### Image processing

FBP and OSEM reconstructions were made with a filter equivalent to a Butterworth (order 10 and cut-off frequency 0.4 cycle/pixel). The resting gated SPECT images were analyzed using the 1-harmonic phase analysis to measure LV systolic dyssynchrony, including systolic phase standard deviation (SD). The core lab evaluated regions of scar fibrosis and indicated the last viable LV region to contract for concordant LV lead placement analysis.

#### Nuclear core lab processing

Quality control was performed related to acquisition count density and gating as well as adequacy of reconstruction. ECTb4 was used to automatically measure left ventricular end-systolic volume (LVESV), left ventricular end-diastolic volume (LVEDV), LVEF, phase SD and site of latest mechanical activation for both the baseline study and 6-month follow-up study. All processing was quality controlled after automatic processing to confirm correct determination of LV oblique angle, base, apex, and LV center cursor position. The site of latest mechanical activation was determined using the 6-segment model (septal, anteroseptal, anterior, lateral, posterior, and inferior). Regions of interest corresponding to the 6-segment model were automatically placed on the 3-dimensional phase distribution (polar map). Each region of interest covers 45° and 6 short axis slices starting from the middle slice towards the base. As there is one myocardial sample per 9°, each region of interest will contain 30 (5 × 6) samples. The mean phases of the 6 segments were calculated by averaging the phases of their 30 samples and then compared. The latest mechanically activated segment was the one with the largest average phase angles. The LVESV, LVEDV, LVEF, phase SD and site of latest mechanical activation for both the baseline study and 6-month follow-up study were reported via SharePoint website using the nuclear core lab analysis form.

#### CRT protocol

Patients were selected for CRT implantation. To assess LV lead position, images were recorded on fluoroscopy using 40° left anterior oblique orientation with caudal tilt and 30° right anterior oblique position to achieve the best separation of the coronary sinus veins.

#### Determination of lead position

In all patients, the final position of the LV lead was determined and categorized as either basal-or-mid, or apical in the anterior, lateral, posterior, inferior and (unlikely) septal or anteroseptal. Lead placement was classified as: concordant (in the last segment), adjacent (up to one segment of the last) and discordant (more than one segment from the last).^17^

## Statistical analysis

Categorical variables were presented as absolute numbers (percentage), and continuous variables were presented as mean and SD or median and interquartile range, according to data normality. The Shapiro-Wilk test was used to analyze data distributions. Comparisons between pre- and post-CRT were performed using Student’s t test for paired samples or Wilcoxon’s test. P < 0.05 was adopted as statistical significance. All analyses were performed using SPSS version 20.0 (IBM Corp., NY, USA).

## Results

After inclusion, 2 patients died of non-cardiological causes, before completing the assessment steps, and 18 participants completed the protocol. Participants’ characteristics are presented in Table 1.

**Table 1:** – Participants’ characteristics

Variables	Patients (n = 18)
Age (years)	65 ± 7
Height (m)	1.36 ± 0.09
Weight (kg)	70.65 ± 16.45
Coronary artery disease. n (%)	8 (44.4)
Diabetes. n (%)	7 (38.9)
**Race. n (%)**	
White	5 (27.8)
Black	9 (50.0)
Others	4 (22.2)

*Data are presented as mean ± standard deviation or number of patients (percentage).*

At baseline, most patients were classified as NYHA III (n = 12), followed by NYHA IV (n = 5) and NYHA II (n = 1). After CRT, 11 out of 18 patients were reclassified to a lower degree of functional limitation, and none of the patients were classified as NYHA IV. All 5 patients classified as NYHA IV and 7 of patients classified as NYHA III pre-CRT were reclassified as NYHA II post-CRT. In addition, patients’ quality of life was improved post-CRT (Figure 1). In our sample, 44.4% of the patients had an ischemic etiology; however, fibrosis burden was below 40%.

**Figure 1:** – Quality of life pre- and post-cardiac resynchronization therapy (CRT). a.u: absolute unit.

The electrocardiogram and SPECT variables are presented in Table 2. CRT increased LVEF, end-diastolic eccentricity (EDE), and end-systolic eccentricity (ESE). In contrast, significant reductions were observed in QRS duration, PR interval, end-diastolic shape index, end-systolic shape index, stroke volume, and myocardial mass post-CRT. Additionally, after CRT, end-diastolic volume index was also reduced.

**Table 2:** – Electrocardiogram and SPECT variables

Variables	Pre-CRT	Post-CRT	p
QRS duration (ms)	194.83 ± 23.68	119.78 ± 11.65	< 0.001
PR interval (ms)	190.0 (167.5 – 210.0)	90.0 (90.0 – 100.0)	< 0.001
EDVi (ml/m^2^)	202.5 (175.0 – 281.75)	164.0 (110.0 – 277.0)	0.058
ESVi (ml/m^2^)	165.5 (124.0 – 209.25)	108.0 (61.5 – 242.75)	0.157
EDSi	0.87 ± 0.11	0.76 ± 0.14	0.001
ESSi	0.80 ± 0.08	0.71 ± 0.12	0.004
LVEF (%)	28.11 ± 5.93	40.94 ± 11.09	0.001
EDE	0.5 ± 0.1	0.6 ± 0.2	0.040
ESE	0.6 ± 0.1	0.7 ± 0.1	0.041
SV (ml)	66.50 (54.75 – 83.75)	51.00 (48.75 – 61.00)	0.002
PBW (degree)	58.5 (39.5 – 108.3)	68 (65 – 72.25)	0.777
PSD (degree)	23.5 (13.4 – 43.1)	21.1 (20.0 – 26.6)	0.372
SPECT phase peak	120.33 ± 34.25	118.22 ± 28.19	0.836
SPECT phase skewness	3.00 (2.86 – 3.37)	2.95 (2.33 – 3.45)	0.231
SPECT phase kurtosis	10.61 (8.05 – 14.42)	8.76 (5.83 – 17.50)	0.586
Myocardial mass (g)	207.5 (185.0 – 262.5)	143.5 (137.25 – 208.25)	0.004

*Data are presented as mean ± standard deviation or median (25^*th*^ – 75^*th*^ percentiles). EDE: end-diastolic eccentricity, EDSi: end-diastolic shape index, EDVi: end-diastolic volume index, ESE: end-systolic eccentricity, ESSi: end-systolic shape index, ESVi: end-systolic volume index, LVEF: left ventricular ejection fraction, PBW: phase bandwidth, PSD: phase standard deviation, SV: stroke volume.*

Analyzing scintigraphic variables after CRT, an increase in SPECT diastolic phase kurtosis was observed. The other variables were unchanged post-CRT.

ESE and EDE are depicted in Figure 2. The CRT LV lead was positioned concordant, adjacent, and discordant in 11 (61.1%), 5 (27.8%), and 2 (11.1%) patients, respectively. ESE and EDE increased post-CRT (Figure 2A and 2C, respectively). Individual data showed that ESE (Figure 2B) and EDE (Figure 2D) increased in both adjacent and concordant positioning. In contrast, these variables decreased only in the 2 patients in whom CRT LV lead position was discordant relative to the last segment to contract.

**Figure 2:** – Box plot and individual data depicting comparisons between pre- and post-CRT (panels A and C) considering whether CRT left ventricle lead position was adjacent, concordant, or discordant (panels B and D). Box plot shows the median (horizontal line within the box), minimum, 25th – 75th percentiles, and maximum. CRT: cardiac resynchronization therapy, EDE: end-diastolic eccentricity, ESE: end-systolic eccentricity.

Figure 3 displays the histogram bandwidth (HBW) in the phase analysis before CRT, demonstrating important dyssynchrony.

**Figure 3:** – Patient who was 64 years old before CRT with heart failure of hypertensive origin and severe LV dysfunction. EF: 14%, EDE: 0.56, ESE: 0.59, ESSi: 0.81, EDSi: 0.83, LVESV: 175 ml, LV mass: 191 g. CRT: cardiac resynchronization therapy, EDE: end-diastolic eccentricity, EDSi: end-diastolic shape index, EF: ejection fraction, ESE: end-systolic eccentricity, ESSi: end-systolic shape index, LV: left ventricular, LVESV: left ventricular end-systolic volume.

Figure 4 displays the images of device implantation procedure at the place of greatest delay.

**Figure 4:** – Fluoroscopy during device implantation (CRT-ICD). The left image shows venography of the coronary sinus and its tributaries and polar map. The right image shows the final positioning of the shock electrodes (right ventricular), right atrium and left ventricle lead in concordant segment, positioned in the coronary sinus.

In Figure 5, a patient demonstrates super-response at 6 months after CRT.

**Figure 5:** – The patient shown in Figure 3, after CRT showing super-response. EF: 57%, ESE: 0.84, EDE: 0.80, ESSi: 0.54 EDSi: 0.6, LVESV: 28 ml, LV mass: 90 g. CRT: cardiac resynchronization therapy, EDE: end-diastolic eccentricity, EDSi: end-diastolic shape index, EF: ejection fraction, ESE: end-systolic eccentricity, ESSi: end-systolic shape index, LV: left ventricular, LVESV: left ventricular end-systolic volume.

## Discussion

LV dyssynchrony has been evaluated by several cardiovascular imaging modalities including echocardiogram with tissue Doppler or even strain^18,19^ or magnetic resonance;^20^ and nuclear imaging through radioisotopic ventriculography or SPECT.^21^ LV contraction was initially successfully analyzed by radionuclide ventriculography, but, with the addition of phase analysis to gated SPECT and its subsequent validation, this technique has been shown to have excellent potential in determining LV mechanical dyssynchrony. Gated SPECT allows the assessment of LV dyssynchrony using Fourier harmonic functions to estimate wall thickening throughout the cardiac cycle and to determine the regional onset moment of mechanical contraction of the ventricle, obtaining a three-dimensional quantitative analysis of the entire LV.

Phase analysis of myocardial perfusion scintigraphy uses two main variables to predict CRT response. The cutoff values of 135º for band widening (HBW) and 43º for phase SD can predict the clinical response.^8,21,22^ In the present study, we found a mean of 92.5º and 31º before resynchronization for HBW and phase SD, respectively.

In addition to dyssynchrony parameters, phase analysis makes it possible to evaluate the last LV segment to contract in a highly reproducible manner. Patients with left bundle branch block tend to have onset of mechanical LV contraction earlier in the cardiac cycle in the septal wall and later in other regions of the myocardium because of the deceleration of the propagation of the nerve impulse by the conduction system, causing a late activation, with the last site of contraction most commonly located on the inferior or lateral wall.^9^ Studies that performed LV lead implantation in agreement with the findings of the last segment to contract by the gated-SPECT phase analysis demonstrated significant clinical improvement.^9^ The parameters that indicate acute change in synchronism after CRT are: (a) presence of baseline dyssynchrony defined by SD and HBW > 2 SD above normal limits, (b) presence of fibrosis < 40% of the LV and (c) agreement of the electrode position, defined as the placement of the LV electrode in the last segment to contract, based on the polar map.^10,23^ This variable was feasible in the present study, where the implantation of the electrode in agreement with the last segment to contract was achieved in approximately 60% of the patients.

According to previous findings, eccentric LV hypertrophy is an independent variable for arrhythmogenic sudden cardiac death,^24^ a variable present in all patients in the study, added to LVEF below 35%, as well as 44% of participants with coronary artery disease.

As observed in a previous study, most patients in this study (n = 17, 94.4%) presented for resynchronization therapy in NYHA functional class III or IV.^25^ The findings of improved quality of life, assessed using the Minnesota Questionnaire were significant. Clinical functional assessments in the present study corroborated the benefit of CRT already observed in several studies.^25-27^ Despite evaluating subjective data, the MLHFQ refers to the patients’ perception of their symptoms, and the InSync study described this perception of improvement assessed by the MLHFQ.^28^ Nascimento et al. demonstrated improved quality of life and a relationship between electromechanical synchronism and response to CRT in the position of the LV lead guided by gated SPECT.^29^

Our findings are in agreement with the data by He et al.,^30^ where parameters of the LV geometry obtained by gated SPECT were able to predict super-response to CRT associated with the orientation of the LV electrode placement by gated SPECT, in which we observed a significant change of the variable LV eccentricity in both LV systole and diastole, denoting reverse remodeling after CRT guided by gated SPECT. A significant finding in our data is that, in the discordant position of the LV electrode in 2 patients, the eccentricity variable behaved differently from the other positions, not leading to reverse remodeling, with worsening of LV geometry.

Recent studies have reported the presence of perfusion defects or scar tissue and the influence on the response to CRT,^31,32^ and gated SPECT has an advantage in this approach, because it makes it possible to integrate assessment of LV function, perfusion (to identify ischemia and scar tissue), and dyssynchrony.

Follow-up of the Imaging CRT study that used multimodality to guide LV lead implantation in CRT showed no reduction in the composite outcome of HF hospitalizations and all-cause mortality.^33^ The classification of LV remodeling based on relative wall thickness and ventricular mass leads us to the concept that, in eccentric remodeling, there is dilation of the LV chamber without an increase in LV mass.^34^

Data from the MIRACLE study of sustained reverse remodeling demonstrated a sustained reduction in ventricular mass at 6 and 12 months after CRT. As observed in our data, myocardial mass had a significant reduction in the pre- and 6 months post-CRT analysis, 207.5 g and 143.5 g, respectively.^35^

The present study has some limitations, including the following: the inclusion of a relatively small number of patients; non-randomization of the patients to a control group; short follow-up period, which may influence the evaluation of the behavior of ventricular remodeling variables; and non-use of quadripolar electrodes, which could expand the assessment of LV electrode placement.

Future randomized studies with a greater number of patients are needed to better assess correlation between LV lead implantation and ventricular remodeling in CRT.

## Conclusion

LV lead implantation in CRT guided by gated SPECT scintigraphy is feasible. The placement of the electrode concordant or adjacent to the last segment to contract was a determinant of response to CRT, leading to reverse remodeling assessed by the LV eccentricity index, and this placement was achieved in 88.9% of the patients in the present study. Future prospective studies are needed in larger populations.
